# Elements of Materia Medica and Therapeutics

**Published:** 1846-12

**Authors:** 


ARTICLE XI.

*Elements of Materia Medica and Therapeutics.* By EDWARD BALLARD, M. D., London, Physician to the St. Pancras Royal General Dispensary, and Medical Tutor in University College, London, and ALFRED BARING GARROD, M. D., London, Physician to the Fore Street Dispensary, and Lecturer on Materia Medica and Therapeutics in the Aldersgate School of Medicine. With additions and alterations by R. EGLESFIELD GRIFFITH, M. D. Philadelphia: Hogan & Thompson. 1846. From the publishers. (For sale by Brautigam & Keen, Chicago.)

This work is designed to be strictly elementary, and so far as the history of drugs is concerned, a compilation. In our large works on Materia Medica there is much matter not absolutely necessary for the practitioners to know, and is scarcely ever read attentively by the medical student. In reference to this work, the authors say, "without depreciating then, the larger works in our language as books of reference, the present is intended to be one, every word of which the student ought to read, and with whose entire contents he should render himself familiar."

The first division of the work treats of the general principles of therapeutics, and of chemistry and natural history as applied to materia medica, occupying 67 pages. The second is devoted to the consideration of the individual articles of the *materia medica.* A chemical classification is adopted for the inorganized, and a natural historical one for the organized substances.

That portion of the work including the general principles of therapeutics, chemistry, and natural history as applied to materia medica, and the inorganic medicinal agents, was prepared by Dr. Ballard; and the remainder, including the organic medicines, by Dr. Garrod.

The American editor has added the formulæ of the U. S. pharmacopœia, and also some medicinal plants indigenous to this country, and extended or modified the description of others. In an appendix are added some of the more recent pharmaceutical preparations, and the therapeutical agents, electricity, bathing, mineral waters, blood letting, &c.

The work is well calculated for what it purports to be, viz: an elementary work on materia medica. In addition to what is original, those main and leading facts which are of the utmost importance to the physician, have been gathered from standard works, from among other matters of less importance.

The matter and arrangements are good. The latest improvements in medicines are noticed, and much has been condensed within a small compass, which renders it a valuable text book for medical students. J. McL.

